# Tubular proteinuria due to hereditary endocytic receptor disorder of the proximal tubule: Dent disease and chronic benign proteinuria

**DOI:** 10.1007/s00467-025-06745-x

**Published:** 2025-03-31

**Authors:** Nana Sakakibara, Kandai Nozu

**Affiliations:** Department of Pediatrics, Kobe University Graduate School of Medicine, 7-5-1 Kusunoki-Cho, Chuo-Ku, Kobe, 650-0017 Japan

**Keywords:** Tubular proteinuria, Megalin, Cubilin, Dent disease, Chronic benign proteinuria, PROCHOB

## Abstract

The proximal tubule has a highly efficient endocytic pathway dedicated to reabsorbing albumin and low-molecular–weight proteins that have passed through the glomerular filtration barrier. This pathway is dependent on multi-ligand receptors: megalin and cubilin. Abnormalities in genes associated with endocytosis in the proximal tubule can lead to tubular proteinuria, where the urine contains albumin and low-molecular–weight proteins. Dent disease is a hereditary X-linked disorder characterized by low-molecular–weight proteinuria, hypercalciuria, nephrocalcinosis, nephrolithiasis, and progressive kidney dysfunction, often leading to CKD stage 5. *CLCN5* is the gene responsible for Dent disease-1 and encodes the voltage-gated chloride channel ClC-5. Meanwhile, *OCRL* is the causative gene of Dent disease-2 and encodes phosphatidylinositol 4,5-bisphosphate 5-phosphatase, and its variants are also associated with Lowe syndrome. ClC-5 and OCRL are essential to the endocytic machinery, and their loss affects endosomal acidification and trafficking, resulting in disruption of megalin and cubilin recycling. *CUBN*, which encodes cubilin, was originally identified as the causative gene of Imerslund–Gräsbeck syndrome, a disorder of megaloblastic anemia associated with proteinuria. However, recently, a biallelic C-terminal variant of *CUBN* was shown to be responsible for isolated proteinuria without kidney dysfunction. This proteinuria is recognized as a new disease concept called chronic benign proteinuria (proteinuria, chronic benign: PROCHOB), which contradicts the common belief that proteinuria is harmful and ultimately leads to kidney damage. This article deepens the understanding of genetic tubular proteinuria and its origins, focusing on the role of megalin- and cubilin-mediated endocytosis in the proximal tubule.

**Graphical abstract:**

**Figure:**
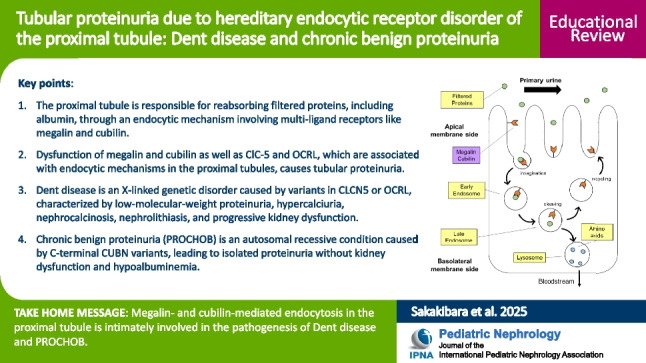
A higher resolution version of the Graphical abstract is available as  Supplementary information

**Supplementary Information:**

The online version contains supplementary material available at 10.1007/s00467-025-06745-x.

## Introduction

The human kidney consists of approximately one million functional units known as nephrons, which can be divided into two main parts: the glomerulus, responsible for filtering plasma to produce what is referred to as “primary” urine, and the tubule, which reabsorbs the majority of this primary urine.

The origin of renal proteinuria can be traced to either the glomerular filtration system or the proximal tubular reabsorption process. The glomerular filtration barrier acts as an obstacle to protein filtration, preventing protein leakage into urine; however, it is not a complete filtration barrier, as a significant amount of albumin and low-molecular–weight proteins are filtered into the primary urine. The sieving coefficient quantifies the membrane’s selectivity and efficiency in filtering out substances, reflecting how easily a substance can pass through the membrane. It specifically refers to the ratio of the concentration of a substance in the filtered liquid to its concentration in the liquid before filtration. Assuming a plasma albumin concentration of 4 g/dL, a glomerular filtration rate of 100 mL/min, and a glomerular sieving coefficient of 0.001–0.003 [1], the amount of albumin that filters through the glomerulus was calculated to be approximately 6–18 g/day (100 mL/min × 60 min × 24 h × 4 g/100 mL × 0.001–0.003). In healthy individuals, most of these proteins are reabsorbed in the proximal tubules, resulting in only a small amount being excreted in the urine. Megalin and cubilin/amnionless complex are expressed in the proximal tubules and play a crucial role in this reabsorption mechanism [2, 3].

This article focuses on Dent disease and chronic benign proteinuria (proteinuria, chronic benign: PROCHOB), which result in low-molecular–weight proteinuria due to malfunctioning endocytic machinery.

### Protein reabsorption mechanism in the proximal tubule (endocytosis)

The proximal tubule possesses a highly efficient endocytic pathway specialized in retrieving albumin and low-molecular–weight proteins that are filtered out by the glomerular filtration barrier. This process relies on multi-ligand receptors, megalin and cubilin/amnionless (CUBAM) complex, to facilitate the uptake of filtered ligands. Both are expressed in the lumen of the proximal tubule and they interact with each other. Most of the plasma proteins that pass through the glomerular filtration barrier are reabsorbed in the proximal tubule, particularly in the S1 segment, through endocytosis [4]. Megalin and cubilin bind to a variety of different ligands including vitamins, iron carriers, hormones, enzymes, and immune-related proteins. Some ligands are specific to either megalin or cubilin, while others are shared by them both [5] (Table 1).

**Table 1:** The ligands for megalin and cubilin

Megalin	Cubilin	Both of them
**Vitamin carrier proteins**		
Transcobalamin–vitamin B12 Retinol-binding protein Folate-binding protein	Intrinsic factor vitamin B12	Vitamin D–binding protein
**Other carrier proteins**		
Lactoferrin Selenoprotein P Metallothionein Neutrophil gelatinase–associated lipocalin Odorant-binding protein Transthyretin Liver-type fatty acid–binding protein Sex hormone–binding globulin	Transferrin	Albumin Myoglobin Hemoglobin
**Lipoproteins**		
Apolipoprotein B Apolipoprotein E Apolipoprotein J/clusterin Apolipoprotein H Apolipoprotein M	Apolipoprotein A-1 High-density lipoprotein	
**Hormones and signaling proteins**		
Parathyroid hormone Insulin Epidermal growth factor Prolactin Thyroglobulin Sonic hedgehog protein Angiotensin II Leptin Bone morphogenic protein 4 Connective tissue growth factor Insulin-like growth factor Survivin	Fibroblast growth factor	
**Enzymes and enzyme inhibitors**		
Plasminogen activator inhibitor type I Pro-urokinase Lipoprotein lipase Plasminogen α-Amylase Lysozyme Cathepsin B α-Galactosidase A Cystatin C		Recombinant activated factor VIIa
**Immune- and stress-related proteins**		
Pancreatitis-associated protein 1 β2-Microglobulin	Clara cell secretory protein	Immunoglobulin light chains α1-Microglobulin

The endocytosis in the proximal tubule appears to occur primarily via the clathrin-mediated endocytic pathway. During endocytosis, receptors bind and internalize many ligands, after which small invaginations of the plasma membrane are created, containing receptors and ligands. These invaginations then separate from the membrane to form endocytic vesicles, which transport the contents to the sorting endosomal compartment. From the early endosome compartment, the internalized material is directed to the lysosomal compartment through late endosomes. Dissociation of the ligand from the receptor occurs along the endocytic pathway. This process is mediated by a basket-like coat primarily made up of clathrin. After the endocytic vesicles are released from the plasma membrane, the clathrin coat is degraded and its components are shed and recycled for use by new endocytic vesicles.

**Fig. 1:**
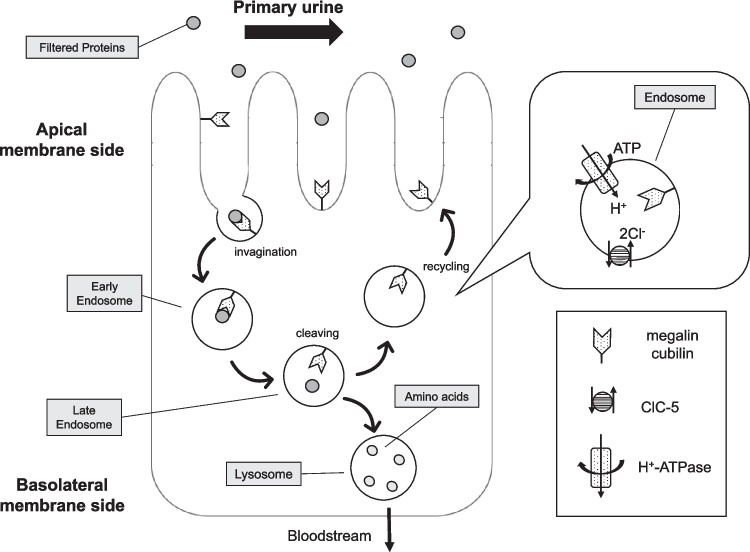
Mechanisms of protein reabsorption in the proximal tubule. The filtered proteins, namely, low-molecular–weight proteins and albumin, bind to megalin and cubilin expressed on the apical membrane of the proximal tubular cells. Once internalized, endocytic vesicles containing ligand–receptor in complex are then transported from early endosomes to lysosomes. The endosomal compartments become increasingly acidified, after which ligand–receptor dissociation occurs. In the lysosomal compartment, ligand proteins are cleaved, and the resulting amino acids pass through the basolateral membrane and return to the bloodstream. Meanwhile, the endosome moves back to the apical membrane, and then megalin and cubilin are recycled back to the apical membrane. ClC-5 is a 2Cl^−^/H^+^ exchange transporter in endosomal membranes. This protein colocalizes with H^+^-ATPase and provides an electrical shunt for efficient endosomal acidification

A common mechanism triggering ligand–receptor dissociation is the decrease in pH in each successive endocytic compartment. In the lysosomal compartment, the internalized material is cleaved, and the resulting amino acids exit the cell across the tubular basolateral membrane and return to the bloodstream. In contrast, the endosome moves back toward the tubular lumen, and then megalin and cubilin are recycled back to the luminal side of the tubule (Fig. 1) [6, 7].

### Tubular endocytosis-related proteins associated with genetic disorders

#### Megalin

Megalin, low-density-lipoprotein (LDL) receptor-related protein 2 (LRP2), is a large (600 kDa) transmembrane protein belonging to the low-density-lipoprotein (LDL) receptor family. It is expressed on the apical surface of various absorptive epithelial cells, particularly in the proximal tubule [8, 9], where it acts as a multifunctional endocytic receptor. Donnai–Barrow syndrome (OMIM: 222448), a very rare autosomal recessive disorder, arises from abnormalities in the gene *LRP2* that encodes megalin. This syndrome is known to present characteristic facial features, ocular hypertelorism, severe myopia, sensorineural hearing loss, developmental delay, agenesis of the corpus callosum, congenital diaphragmatic hernia, and umbilical or inguinal hernia [10, 11]. All reported cases have shown low-molecular–weight proteinuria, with several instances of progressive kidney dysfunction and focal segmental glomerulosclerosis [12].

#### Cubilin

Cubilin is also a large (460 kDa) endocytic receptor essential for intestinal vitamin B12 uptake and for protein (e.g., albumin) reabsorption from the kidney filtrate [13, 14]. Since cubilin is not a transmembrane protein itself, it forms a complex called CUBAM with the transmembrane protein amnionless, allowing it to be anchored to the apical membrane and contribute to the reabsorption. Loss of function of either cubilin or amnionless has been shown to cause autosomal recessive vitamin B12 malabsorption syndrome, also known as Imerslund–Gräsbeck syndrome (IGS) (OMIM: 261100). The disease involves megaloblastic anemia due to severe B12 deficiency and proteinuria [15]. Biallelic *CUBN* variants can cause isolated proteinuria without the megaloblastic anemia seen in IGS, which is termed PROCHOB (OMIM: 618884). Research using knockout mice has shown that the presence of cubilin is essential for the reabsorption of albumin, and megalin is also thought to indirectly participate in albumin reabsorption by promoting internalization of the cubilin–albumin complex [16].

#### ClC-5

Endosomal acidification occurs through the coordinated action of ClC-5 channels, which provide an electrical shunt, and H⁺-ATPase, which pumps protons into endosomes. ClC-5, encoded by *CLCN5*, is a member of the voltage-gated chloride channel (ClC) family and expressed in the proximal tubule, thick ascending limb, and collecting duct [17]. This kidney-specific channel plays a key role in the receptor-mediated endocytic pathway in the proximal tubule, functioning as a 2Cl^−^/H^+^ exchange transporter in endosomal membranes. This protein colocalizes with the H^+^-ATPase in intracellular vesicles and is thought to provide an electrical shunt for efficient vesicle acidification during endocytosis. For the H^+^ATPase to pump protons into the endosome, charge balance must be maintained. ClC-5 allows Cl⁻ to enter the endosome, helping maintain the electrochemical equilibrium across the membrane and enhancing proton pump efficiency. Without this chloride influx, a positive charge would build up and hinder proton influx, making further acidification difficult [18–20] (Fig. 1). In Dent disease-1 (OMIM: 300009), the reduced function of ClC-5 leads to impaired acidification of endosomes and/or a decrease in chloride concentration, resulting in delayed maturation of early endosomes and dysfunction of endosomal recycling. As a consequence, the expression of endocytic receptors in the tubular lumen decreases, and the endocytosis of low-molecular–weight proteins is impaired [21–23]. In fact, it has been reported that megalin levels are reduced in both urine and kidney tissue in patients with Dent disease-1 [24, 25].

#### OCRL

OCRL is a phosphatidylinositol 4,5-bisphosphate [PI(4,5)P2] 5-phosphatase that dephosphorylates phosphoinositides, which is encoded by *Oculocerebrorenal syndrome of Lowe* (*OCRL*). *OCRL* was originally described as the gene responsible for Lowe syndrome (OMIM: 309000), a condition characterized by congenital cataracts, Fanconi syndrome, muscle weakness, and psychomotor developmental delay [26–28]. *OCRL* was later identified as the second causative gene of Dent disease-2 (OMIM: 300555) [29]. OCRL associates with various subcellular compartments including clathrin-coated vesicles, early endosomes, the trans-Golgi network, and the primary cilium, which appear to regulate many processes within the cell involved in endosomal transport, most of which depend on the coordination of membrane dynamics and remodeling of the actin cytoskeleton [30, 31]. In Lowe syndrome and Dent disease-2, trafficking of endocytic receptors from early endosomes to the plasma membrane occurs less efficiently. The loss of OCRL impedes the dephosphorylation of PI(4,5)P2, leading to its local accumulation, which is implicated in a failure to break apart (uncoat) clathrin-coated vesicles, resulting in aberrant actin polymerization [30, 32, 33]. As a result, receptors accumulate in endosomes and are incorrectly sorted to lysosomes instead of being recycled to the apical membrane [30, 34].

#### EHD1

Recently, a homozygous variant of *EHD1* (p.R398W) was identified in six patients with low-molecular–weight proteinuria similar to Dent disease and sensorineural hearing deficit. Functional analyses using mouse and zebrafish models also revealed similar symptoms. *EHD1* encodes EH domain containing 1, the ciliary-associated protein expressed in endosomes and the Golgi apparatus, and this protein is also known to be involved in endosomal recycling [35, 36].

### Dent disease

#### Molecular genetics

Dent disease is an X-linked hereditary tubular disorder, the causative genes of which are *CLCN5* and *OCRL*. Approximately 60% of clinically diagnosed cases of Dent disease are Dent disease-1 and around 15% are Dent disease-2, while in the remaining 20–25%, no genetic abnormalities are identified [37]. The disease primarily affects males, and female carriers usually have milder symptoms [38] or are asymptomatic. However, there are a few affected females who present with symptoms similar to those of affected males. Skewed X-chromosome inactivation may be one of the factors associated with phenotypic diversity in female patients with Dent disease [39, 40]. Family history can be helpful for diagnosis, but sporadic cases do occur.

Although no clear genotype–phenotype correlation has been observed in Dent disease-1 [38, 41], truncated variants appear to be more frequently associated with kidney failure than non-truncated ones [42]. There also appears to be a difference in the domains where variants are clustered between truncating and non-truncating variants [42, 43].

**Fig. 2:**
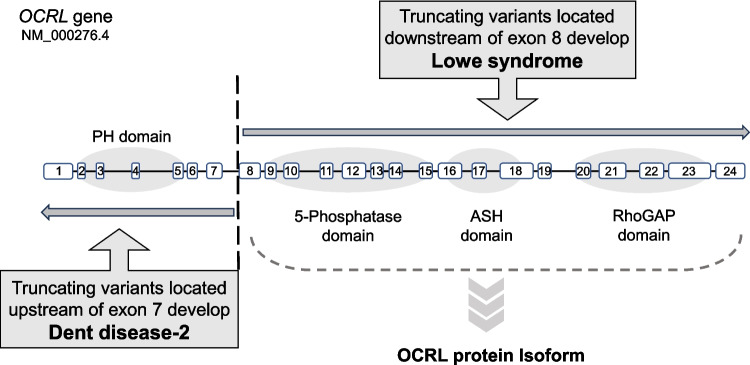
Genotype–phenotype correlation in Lowe syndrome with truncating variants. Truncating variants of Lowe syndrome are present only in exons 1–7 in Dent disease-2 and only in exons 8–24 in Lowe syndrome, and the 5-phosphatase domain is located in the region encoded downstream of exon 8. This led to the understanding that there is an OCRL isoform

A review of cases with *OCRL* variants showed that truncating variants were present only in exons 1–7 in Dent disease-2 and only in exons 8–24 in Lowe syndrome, and the 5-phosphatase domain is located in the region encoded downstream of exon 8. This led to the understanding that there is an important splice variant (i.e., OCRL isoform) that maintains OCRL function, and this isoform rescues Dent disease-2 from systemic OCRL dysfunction in Lowe syndrome [44, 45] (Fig. 2). Indeed, an *OCRL* transcript variant starting from exon 6 has been identified, from which a functional “isoform” protein with a start codon at exon 8 is synthesized [46].

#### Clinical manifestations and epidemiology

Dent disease, whose primary pathogenesis is endocytic disorder in the proximal tubules, is known to present with low-molecular–weight proteinuria, hypercalciuria, nephrocalcinosis, and nephrolithiasis, and it gradually progresses to CKD stage 5 [37].

Low-molecular–weight proteinuria is a hallmark of Dent disease, with urinary β2-microglobulin, α1-microglobulin, or retinol-binding protein levels being 100 to 1000 times higher than normal [47]. Proteinuria (albuminuria) can reach nephrotic levels, but it typically does not accompany hypoalbuminemia or edema. Hypercalciuria, nephrocalcinosis, and nephrolithiasis are typical signs of Dent disease, although some of the cases do not present with these features and instead present with isolated nephrotic-range proteinuria with focal segmental and/or global glomerulosclerosis [48]. Alterations in endosomal function and parathormone endocytosis affect calcium and phosphate transport in the proximal tubules, suggesting an association with hypercalciuria and hypophosphatemia in Dent disease [7].

Additionally, patients may exhibit an incomplete form of Fanconi syndrome, such as glucosuria, aminoaciduria, hypophosphatemia, and rickets, but acidosis is rare [49]. Hypokalemia is an occasional feature of Dent disease and is more common in older patients [41]. Interestingly, some cases present with atypical features such as hypokalemic metabolic alkalosis, resembling Bartter-like syndrome [50, 51]. Microhematuria is common in patients with Dent disease [52] and is thought to be due to impaired hemoglobin reabsorption.

It was suggested that 30–80% of cases progress to CKD stage 5 by the age of 30–50 years [53]; however, in some cases, CKD stage 5 may not develop until later in life. The reason why Dent disease presents with progressive kidney dysfunction is not well understood. However, kidney biopsy tissue from patients with Dent disease has shown inflammation and fibrosis in the tubular interstitium, as well as glomerular sclerosis and partial loss of podocyte foot processes. It has also been reported that the proportion of sclerotic glomeruli in Dent disease increases with age at the time of biopsy [54]. Much about the kidney damage in Dent disease remains unknown, although it is known that there is no correlation between kidney failure and nephrocalcinosis [47, 52]. First, it is unclear whether glomerular sclerosis occurs as a consequence of tubular damage or if it is directly related to podocyte dysfunction. However, given that ClC-5 is expressed in human podocyte foot processes [55] and ClC-5 loss may alter podocyte function either through cytoskeletal disorganization due to abnormal actin structure or through impairment of nephrin recycling [56], it seems possible that podocyte dysfunction is directly associated with the kidney dysfunction.

The clinical manifestations of Dent disease-1 and Dent disease-2 are not exactly the same. In Dent disease-1, kidney symptoms are typically the only manifestations, whereas some cases of Dent disease-2 may present with extrarenal manifestations, including short stature, cataracts, and elevated muscle enzymes (AST/ALT, CK, LDH) [57, 58]. Lowe syndrome presents with severe symptoms such as congenital cataracts, Fanconi syndrome, hypotonia, and global developmental delay, whereas Dent disease-2 often does not exhibit noticeable extrarenal symptoms and is clinically much milder than Lowe syndrome.

There also seems to be a difference in kidney symptoms between the two disease types, such as hypercalciuria being more common and nephrocalcinosis being less common in Dent disease-2 than in Dent disease-1 [58]. In a large cohort study in France, no significant influence of the genotype of Dent disease-1 or Dent disease-2 on the rate of glomerular filtration rate decline was observed [41]. Meanwhile, Dent disase-2 has been reported to be associated with a higher risk of kidney dysfunction and CKD stage 5 [52, 58].

The prevalence of Dent disease is unknown, and there are likely many undiagnosed cases [48, 53]. No populations known to be at particular risk of this disease have been identified.

#### Treatment

There is currently no specific treatment for Dent disease, and no evidence-based symptomatic therapy has been established. Non-pharmacological therapy, such as adequate fluid intake to prevent stone formation and salt restriction to correct hypercalciuria, is reasonable. However, especially in pediatric cases, it is not uncommon for the condition to be monitored without treatment.

Thiazide diuretics and renin–angiotensin system (RAS) inhibitors should be used with caution in Dent disease patients [59]. Reports have suggested that the administration of thiazide diuretics is effective in reducing urinary calcium excretion, but they are associated with adverse events such as hypovolemia and hypokalemia [60, 61]. No significant effect of the use of RAS inhibitors on reducing proteinuria was observed [41, 62], but the risk of hypotension and hypovolemia was also noted [62, 63]. However, whether thiazide and RAS inhibitors help to slow the decline in kidney function has not been investigated.

Although conducted only on mice, in one study, citrate supplementation in ClC-5-knockout mice was found to delay the progression of kidney failure [64].

Research on the treatment of Lowe syndrome and Dent disease-2 has also been performed, focusing on the abnormal actin polymerization in OCRL deficiency. In this research, the PI3K inhibitor alpelisib suppressed aberrant actin polymerization by reducing levels of PI(4,5)P2 and PI(3)P, causing endocytosis defects in proximal tubules, increased megalin expression in the kidneys and reduced low-molecular–weight proteinuria and albuminuria in a humanized mouse model for Lowe syndrome/Dent disease-2. Alpelisib is already a safe treatment approved for other diseases, and its use for treating these conditions is also highly anticipated [65].

### Chronic benign proteinuria (proteinuria, chronic benign: PROCHOB)

*CUBN* is known to be the causative gene of Imerslund–Gräsbeck syndrome, which is often associated with proteinuria [66]. A homozygous *CUBN* variant was first detected in two siblings with isolated proteinuria in 2011 [67]. Furthermore, in 2020, it was reported that, in a cohort of European patients with proteinuria, biallelic *CUBN* variants on the C-terminal side of the vitamin B12 binding site were not associated with Imerslund–Gräsbeck syndrome or kidney dysfunction, despite the presence of proteinuria. Although these individuals exhibited proteinuria, their kidney function remained normal [68]. This finding contrasts with the commonly held belief that proteinuria is harmful and ultimately leads to kidney damage.

This condition has been recognized as a new disease entity, termed PROCHOB. The detailed phenotype of PROCHOB was revealed in subsequent studies. Specifically, the patients show no hypoalbuminemia, no kidney dysfunction, and sub-nephrotic-range proteinuria of approximately 0.5 to 1.5 g/gCr, with a lack of response to RAS inhibitors. However, unlike in Dent disease, urinary β2-microglobulin and α1-microglobulin levels remain normal [68–71], so these urinary findings resemble glomerular proteinuria, but the proteinuria in PROCHOB is actually tubular proteinuria. The patients usually show no remarkable findings on kidney biopsy, but a few reports of PROCHOB with focal segmental glomerulosclerosis have been published [70–72]. It is not known whether focal segmental glomerulosclerosis is secondary to tubular proteinuria or a primary change. However, cubilin is expressed in human glomerular podocytes [73], suggesting that it is a primary change. Distinguishing between glomerular proteinuria and this condition based solely on urinary and pathological findings can be difficult, and a definitive diagnosis currently relies on genetic analysis. Given the clinical course described above, it is important to consider proactive genetic testing to avoid unnecessary treatment interventions or repeated kidney biopsies.

There is a clear correlation between genotype and phenotype associated with *CUBN* variants [68]. All IGS-related variants are located only on the N-terminal side or within the vitamin B12 binding domain [74, 75]. In contrast, PROCHOB is caused by the variants located on the C-terminal side of this region. This genotype–phenotype correlation may be related to the presence of intestinal transcripts truncated immediately after the vitamin B12 binding domain in the Genotype-Tissue Expression (GTEx) database [68, 76].

Several genome-wide association studies have discovered various C-terminal *CUBN* variants associated with the risk of albuminuria [77–81]. It has also been reported that premature truncation of cubilin is more likely to occur downstream of the vitamin B12 binding domain in the normal population. These findings support the association of human C-terminal *CUBN* variants with isolated proteinuria.

## Key summary points

- The proximal tubule is responsible for reabsorbing filtered proteins, including albumin, through an endocytic mechanism involving multi-ligand receptors like megalin and cubilin.
- Dysfunction of megalin and cubilin as well as ClC-5 and OCRL, which are associated with endocytic mechanisms in the proximal tubules, causes tubular proteinuria.
- Dent disease is an X-linked genetic disorder caused by variants in *CLCN5* or *OCRL*, characterized by low-molecular–weight proteinuria, hypercalciuria, nephrocalcinosis, nephrolithiasis, and progressive kidney dysfunction.
- Chronic benign proteinuria (PROCHOB) is an autosomal recessive condition caused by C-terminal *CUBN* variants, leading to isolated proteinuria without kidney dysfunction and hypoalbuminemia.

## Multiple Choice Questions

Answers are given following the reference list.

- 1: Which of the following statements are true? (Select all that apply)
  a The endocytic receptors megalin and cubilin play a crucial role in the reabsorption of proteins in the proximal tubules.
  b Malfunctioning endocytic machinery in the proximal tubules is the main cause of tubular proteinuria in Dent disease and PROCHOB.
  c In healthy individuals, albumin rarely passes through the glomerular filtration barrier.
  d Donnai–Barrow syndrome is caused by an abnormality in *OCRL*, which encodes megalin.
- Question 2: Which of the following statements are true about Dent disease? (Select all that apply)
  a The disease is inherited in an autosomal recessive manner.
  b CKD stage 5 is rare.
  c Hematuria may occur.
  d Calcification of the kidney may be observed.
- Question 3: Which of the following is true about PROCHOB?
  a It shows hypoalbuminemia and kidney dysfunction.
  b It exhibits sub-nephrotic-range proteinuria but normal kidney function.
  c It is caused by variants in the N-terminal side of *CUBN*.
  d It always shows abnormal findings in kidney biopsies.

## Supplementary Information

Below is the link to the electronic supplementary material.

Graphical abstract (PPTX 179 KB)
