# Supplementary material for: Tubular proteinuria due to hereditary endocytic receptor disorder of the proximal tubule: Dent disease and chronic benign proteinuria
Source: Pediatr Nephrol. 2025 Mar 31;40(11):3367–77. doi: 10.1007/s00467-025-06745-x (PMC12484296; doi:10.1007/s00467-025-06745-x)
Supplement: Supplementary file 1 — Graphical abstract (PPTX 179 KB) [file 467_2025_6745_MOESM1_ESM.pptx]

## Slide 1
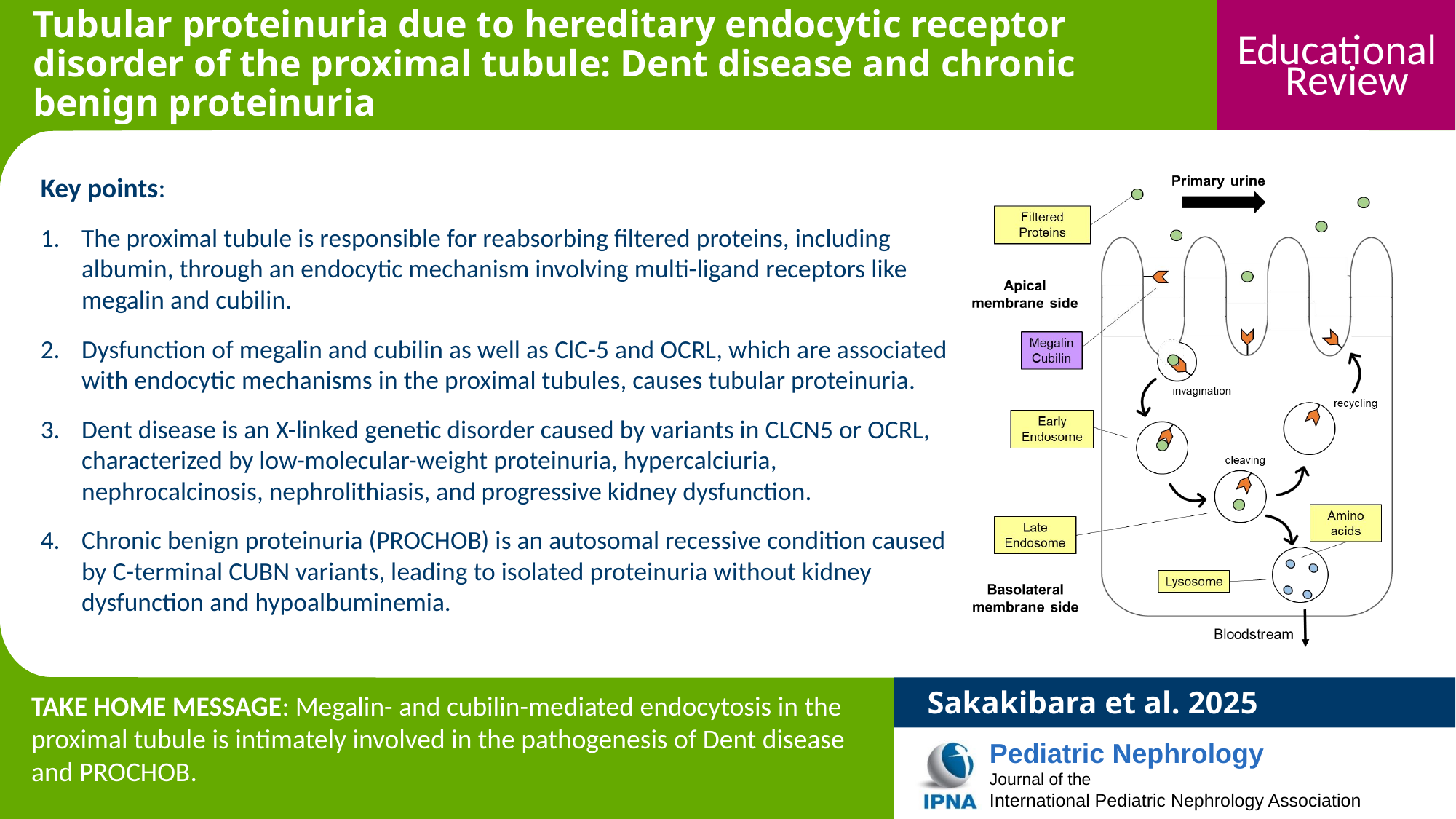

Tubular proteinuria due to hereditary endocytic receptor disorder of the proximal tubule: Dent disease and chronic benign proteinuria
Key points:
The proximal tubule is responsible for reabsorbing filtered proteins, including albumin, through an endocytic mechanism involving multi-ligand receptors like megalin and cubilin.
Dysfunction of megalin and cubilin as well as ClC-5 and OCRL, which are associated with endocytic mechanisms in the proximal tubules, causes tubular proteinuria.
Dent disease is an X-linked genetic disorder caused by variants in CLCN5 or OCRL, characterized by low-molecular-weight proteinuria, hypercalciuria, nephrocalcinosis, nephrolithiasis, and progressive kidney dysfunction.
Chronic benign proteinuria (PROCHOB) is an autosomal recessive condition caused by C-terminal CUBN variants, leading to isolated proteinuria without kidney dysfunction and hypoalbuminemia.
Sakakibara et al. 2025
TAKE HOME MESSAGE: Megalin- and cubilin-mediated endocytosis in the proximal tubule is intimately involved in the pathogenesis of Dent disease and PROCHOB.
